# Supplementary material for: Parameter inference for stochastic biochemical models from perturbation experiments parallelised at the single cell level
Source: PLoS Comput Biol. 2022 Mar 18;18(3):e1009950. doi: 10.1371/journal.pcbi.1009950 (PMC8967023; doi:10.1371/journal.pcbi.1009950)
Supplement: Attachment — Submitted filename: report.pdf [file pcbi.1009950.s002.pdf]

Report on the Manuscript "Parameter inference for stochastic biochemical models from perturbation experiments parallelized at the single cell level" by Davidovic et al.

Summary

The manuscript discusses the inference of parameters for stochastic chemical reaction network models from parallel single-cell trajectories obtained from recent optogenetic experimental methods. These datasets often include large numbers of single-cell trajectories (30 cells as shown in one of the examples) with hundreds of measurement times per cell (240 time points in one of the examples). This complexity poses a challenge to classical methods (Finite State Projection, LNA, SSA) for computing the likelihood function. The authors advanced a new efficient approximation to the likelihood by a clever combination of Kalman filtering, Moment Closure, and Gaussian approximation of the CME's solution. They showed that the new method can provide useful inference of model parameters on parallelized trajectorial data. In addition, the new approximation becomes even more accurate as the measurement frequency increases, which makes it particularly suited to the increased capacity of new experimental methods. The results are very promising, but I think a major revision is needed before the manuscript could be published. There needs to be more discussion and comparison with previous work that also tackles the problem of parameter estimation from trajectorial data. Specific recommendations are given below.

Major comments

1. The manuscript does not cite important previous work that also tackles the problem of parameter inference from single-cell trajectorial data. The idea of coupling particle filtering with approximate or exact simulations of the CME has already been advanced in these papers:

- C. Sherlock, A. Golightly, and D. A. Henderson, "Adaptive, delayed-acceptance MCMC for targets with expensive likelihoods," *J. Comput. Graph. Stat.*, vol. 26, no. 2, pp. 434–444, 2017, doi: 10.1080/10618600.2016.1231064.

- Golightly, D. A. Henderson, and C. Sherlock, "Delayed acceptance particle MCMC for exact inference in stochastic kinetic models," *Stat. Comput.*, vol. 25, no. 5, pp. 1039–1055, 2015, doi: 10.1007/s11222-014-9469-x.

- Golightly and D. J. Wilkinson, "Bayesian parameter inference for stochastic biochemical network models using particle Markov chain Monte Carlo," *Interface Focus*, vol. 1, no. 6, pp. 807–820, 2011, doi: 10.1098/rsfs.2011.0047.

In particular, [Golightly 2015] also used the LNA to approximate the likelihood of single-cell trajectories. In their section 3.2, the authors of that paper also discussed restarting the LNA at intermediate timepoints similarly to the current manuscript. However, I can see that the current method is still novel as it uses moment closure and only "borrows" the Gaussian assumption from the LNA. Still, I think it is important to cite these previous papers and

clearly state how the new method is different from, and more efficient than, the previous applications of LNA.

2. I also want to mention that a variant of the Finite State Projection has also been used to compute the likelihood of single-cell trajectories in the following work:

- Andreychenko, L. Mikeev, D. Spieler, and V. Wolf, "Parameter Identification for Markov Models of Biochemical Reactions," in *Computer Aided Verification*, Berlin, Heidelberg, 2011, pp. 83–98. doi: 10.1007/978-3-642-22110-1_8.

An important similarity is that this work also restarted the CME approximation at each observation time and only used a small efficient FSP approximation that only needed to be valid during the short interval between two observation times. This work should be cited and compared with the new method advanced in the present manuscript.

3. Throughout section 3, the term "LNA" is used to refer to the author's new method which coupled moment closure, Gaussian approximation, and Kalman filtering as defined in Algorithm 1. I find this confusing, and I suggest that the authors use a different name for their method.

4. It is not straightforward to me how The Finite State Projection is used in section 3.1.1. and Figure 2 to compute the single-cell likelihood. Did the authors compute the full dense matrix exponential of the FSP-truncated transition rate matrix? Or was it the much more efficient procedure described in Andreychenko et al. (comment 2 above)? In any case, I recommend a section is added to the supporting information to clearly explain the procedure used to obtain the FSP-based likelihood approximation in Figure 2.
