# Supplementary material for: Parameter inference for stochastic biochemical models from perturbation experiments parallelised at the single cell level
Source: PLoS Comput Biol. 2022 Mar 18;18(3):e1009950. doi: 10.1371/journal.pcbi.1009950 (PMC8967023; doi:10.1371/journal.pcbi.1009950)
Supplement: Attachment — Submitted filename: PointByPointResponse.pdf [file pcbi.1009950.s003.pdf]

Point-by-point response to reviewers

Reviewer #1: The paper by Davidovic et al proposes a method for performing inference of parameters of stochastic reaction networks from time series data. The paper contains a theoretical description of the algorithm as well as extensive validation on simulated data and on one new experimental data set. Overall, the paper is well written and very clear, although cosmetic improvements to the notation could be made.

- We thank the reviewer for the careful assessment and appreciation of our work. In the revised version of the manuscript, we have modified some of the notation. Notably, we have replaced superscripts by subscripts and aligned the notation for vectors with what appears to be more widely used in some of the important references of the paper. If there are further places where the notation is not sufficiently clear, we would be happy to incorporate further changes.

My major reservation though is that I do not understand why the methodology should be considered new. As far as I know, the idea of performing Kalman filtering with an approximation of the transition probabilities for stochastic reaction networks has been initially proposed in an overlooked paper by Ruttor and Opper (Phys. Rev. Lett. 103, 2009) over ten years ago. Several others have followed suit. It is true that several papers use the LNA or similar in an open loop approach, but it is well known that this is simply wrong (see e.g. the popular review by Schnoerr et al in J.Phys.A 2017, sec 6.2-6.4, in particular the incipit of 6.4.1 which very clearly explains how the problem should be formulated in terms of approximating the transition probability in a forward-backward algorithm, which is what the authors of this paper do). Having said that, many of the empirical results of this paper are interesting, e.g. the study of how sampling time affect accuracy, and also and perhaps primarily the real data study. I think the authors should: either be much clearer about the novelty of their method (in case my diagnosis above is wrong), or completely restructure the paper, removing claims of algorithmic novelty and focussing on the analysis and the data part.

- We agree with the reviewer's assessment of the core contributions of our work and note that we have focused abstract, author summary, and claims of novelty entirely on empirical results for new data types and the experimental case study, stating that we "investigate and compare different approaches ... with a particular focus on coupling the linear noise approximation (LNA) or moment closure methods to a Kalman filter." It was not our intention to claim that filtering-based evaluation of likelihoods is a novel idea. The excellent review by Schnoerr et al. formulates the Bayesian measurement update in equation (111) and a calculation of the likelihood in equation (112). We have not found a reference, however, in which general moment closure methods are coupled to an approximation at measurement times that creates a conjugate prior for the likelihood to enable analytical Bayesian updates. The review paper states that moment closure methods are limited to second-order normal moment closure and does not consider that distribution assumptions (e.g. normal) can also be implemented only at measurement times after a different moment closure method has been used to propagate moments through time. We are certainly aware, however, that this is not exactly a major conceptual difference to existing methods. In summary, we fully agree with the reviewer that the inference problem should be formulated as done by Schnoerr et al. and that the contribution of our work is not primarily the method as such but the empirical results on accuracy and computation time in light of the changing data availability as well as the treatment of a real case study.

In response to the comment (and similar comments from other reviewers), we have considered several options for clarifying the core contributions of the paper better. A first option would be to shorten the methodology section of the paper, for instance by referencing existing papers instead of providing equations or by moving the description of the algorithm to the Supporting Information. However, based on the comments received in this review (that the paper is well written and easy to follow), it is our impression that the reviewers considered this section to be useful (this will probably be even more true for less expert readers). Completely restructuring the Introduction does not appear to be a good solution either considering that the first three paragraphs are already largely focused on the implications of novel data types and not on methodology for likelihood evaluation. However, the Introduction of the original manuscript contained only a single sentence that referred to papers in which different approaches for approximate likelihood evaluation had been proposed. Thus, we conclude that what has been missing from the manuscript is a dedicated paragraph where existing methods are referenced before we state what is being studied in our manuscript. In the revised version of our manuscript, we have therefore added such a paragraph to the Introduction (lines 85 to 107). Furthermore, throughout the manuscript, we have removed possibly misleading formulations such as "our approach" for "Algorithm 1" and "classical/standard approach" for "the open loop" method.

Reviewer #2: The manuscript presents an interesting extension of the moment based methods for inferences of parameters governing molecular processes inside single cells. It offers to bypass disadvantages related to a fixed form of transition probabilities by coupling inference to a Kalman filter that is updated over time. The applicability and advantages of the approach are well documented using synthetic and experimental data (opto-genetic gene activation system in E. coli). The text is well written and easy to follow.

- We thank the reviewer for the careful reading and appreciation of our work. In the following, we address the concrete comments point-by-point.

My critique should include to major points:
From my understanding, the different responses of single-cells in section 3.2.3 are assumed to result from noise that is modelled by LNA and Kallman Filter. This is however not necessarily (most likely in my view) the case. Most likely the differences result from differences in copy number of molecular species involved in the system (often referred to as extrinsic noise). If my understanding is correct then treating the data like this is misleading. Several approaches can be easily found that model extrinsic variability.

- This is a very interesting comment that requires a somewhat philosophical discourse as answer. We are quite certain that the reviewer is correct in the assessment that different responses of cells in our data are largely due to noise that is typically referred to as extrinsic. We are also aware of approaches for modeling extrinsic noise in the literature and have ourselves published several methods for parameter inference for models with extrinsic noise (e.g. Zechner et al., PNAS, 2012 and Llamosi et al., PLoS Comput Biol, 2016). The question that needs to be posed to answer the reviewer's comment is what an appropriate interpretation of the terms extrinsic and intrinsic is. From a biological perspective, "extrinsic" typically refers to "extrinsic to the studied

system”, e.g. variability in the number of ribosomes when a specific gene expression process is studied. From a modeling perspective, this would naturally translate to “extrinsic to the model” since we are modeling the studied system after all. However, if we explicitly include extrinsic noise in the model, it obviously stops being extrinsic to the model. In the past, the prevalent interpretation of extrinsic and intrinsic noise in models has therefore been more a question of time scales. Intrinsic is the noise that fluctuates on the time scale of the experiment (presumably this is the time scale of the studied system), extrinsic is the noise from factors that vary between cells but do not significantly change within each cell at the time scale of the experiment (presumably global factors). This is a useful interpretation because it concretely maps to modeling guidelines: intrinsic noise can be represented by a dynamical model and extrinsic noise by constant probability distributions on the model’s parameters. However, a consequence of this is that our interpretation of intrinsic and extrinsic starts to depend on the duration of the experiment: if we were to do a longer experiment, the factors that were previously modeled as extrinsic might start to change and it might not make sense anymore to represent them using constant probability distributions. With the automated microfluidics setups that we are using these days, we can record single-cell processes for several days as opposed to experiments in the past that were typically performed for some hours at the most. In the present manuscript, we have used data from cells that are recorded for one day. This corresponds to several tens of cell generations and we cannot expect that the classical extrinsic factors remain constant at this time scale. We have thus chosen to represent these factors by a dynamically changing (proxy) species (E in the model) instead of using a constant probability distribution on parameters. It is up to the reader (or maybe the scientific community) to decide whether or not E should still be referred to as a model of extrinsic noise. We personally refer to it as time-varying extrinsic noise.
Finally, we note that representing extrinsic variability by a dynamical process implies that the model contains parameters that define the time scale of extrinsic noise fluctuations (parameter h in our model). Inferring such parameters from experimental data can thus inform us if, and how fast, extrinsic noise sources are fluctuating. The reviewer’s comment on extrinsic noise made us realize that we did not sufficiently explain and valorize these results in the original submission of the manuscript. We have therefore added a more detailed discussion on extrinsic noise in Section 3.2 (lines 371 to 376) where the model and the variable E are introduced. Furthermore, we extended the discussion on the particular time scale of extrinsic noise fluctuations that we identified from our data (lines 540 to 543).

I am not sure how novel the ideas to couple Kallman Filter into LNA/Moment based inference is. This should be explained in more detail in the introduction.

- We agree that we have not sufficiently cited the various different approaches that have been developed in the past for parameter inference of time series data. It is undoubtedly true that there exist methods that are conceptually very close to the approach that we use in our manuscript. For us, the main contribution of our manuscript is not the method as such but the analysis of what implications novel data types have for parameter inference and different approaches. This is why we have focused the case studies in the manuscript on working out aspects such as the dependency of the accuracy of likelihood evaluations on the time between measurements and how parameter identifiability depends on the design of the

experiment (diversified inputs). Furthermore, we have focused on practical aspects by providing an experimental case study and analyzing how the computational cost of different methods scales with the number of measured cells, the number of measurement time points, and the number of different perturbations that are used. In response to the reviewer's comment, we have added a dedicated paragraph to the Introduction (lines 85 to 107) where existing methods are discussed before our work is described (see also response to Reviewer #1).

Reviewer #3: The manuscript discusses the inference of parameters for stochastic chemical reaction network models from parallel single-cell trajectories obtained from recent optogenetic experimental methods. These datasets often include large numbers of single-cell trajectories (30 cells as shown in one of the examples) with hundreds of measurement times per cell (240 time points in one of the examples). This complexity poses a challenge to classical methods (Finite State Projection, LNA, SSA) for computing the likelihood function. The authors advanced a new efficient approximation to the likelihood by a clever combination of Kalman filtering, Moment Closure, and Gaussian approximation of the CME's solution. They showed that the new method can provide useful inference of model parameters on parallelized trajectorial data. In addition, the new approximation becomes even more accurate as the measurement frequency increases, which makes it particularly suited to the increased capacity of new experimental methods. The results are very promising, but I think a major revision is needed before the manuscript could be published. There needs to be more discussion and comparison with previous work that also tackles the problem of parameter estimation from trajectorial data. Specific recommendations are given below.

- We thank the reviewer for the careful assessment and appreciation of our work. The specific comments are addressed in the following point-by-point response.

The manuscript does not cite important previous work that also tackles the problem of parameter inference from single-cell trajectorial data. The idea of coupling particle filtering with approximate or exact simulations of the CME has already been advanced in these papers:

• C. Sherlock, A. Golightly, and D. A. Henderson, "Adaptive, delayed-acceptance MCMC for targets with expensive likelihoods," J. Comput. Graph. Stat., vol. 26, no. 2, pp. 434–444, 2017, doi: 10.1080/10618600.2016.1231064.

• Golightly, D. A. Henderson, and C. Sherlock, "Delayed acceptance particle MCMC for exact inference in stochastic kinetic models," Stat. Comput., vol. 25, no. 5, pp. 1039–1055, 2015, doi: 10.1007/s11222-014-9469-x.

• Golightly and D. J. Wilkinson, "Bayesian parameter inference for stochastic biochemical network models using particle Markov chain Monte Carlo," Interface Focus, vol. 1, no.6, pp. 807–820, 2011, doi: 10.1098/rsfs.2011.0047.

In particular, [Golightly 2015] also used the LNA to approximate the likelihood of single-cell trajectories. In their section 3.2, the authors of that paper also discussed restarting the LNA at intermediate timepoints similarly to the current manuscript. However, I can see that the

current method is still novel as it uses moment closure and only "borrows" the Gaussian assumption from the LNA. Still, I think it is important to cite these previous papers and clearly state how the new method is different from, and more efficient than, the previous applications of LNA.

- The reviewer is correct to point out that we have only cited a non-complete selection of papers that used similar approaches. We are aware that much more work has been done on this topic, in particular within the UK statistics community. We also agree with the reviewer's assessment that the presented method differs from other approaches in that it only "borrows" the Gaussian assumption from the LNA. Nonetheless, we believe that the core contribution of the paper is not the method as such but the analysis of what implications novel data types have for parameter inference. This has led to an organization of the abstract, author summary, and introduction that focused on the data perspective and provided only a minimal review of existing methodology. In response to the reviewer's comment, we have added a dedicated paragraph to the Introduction (lines 85 to 107) where existing methods are discussed before our work is described (see also response to Reviewer #1). The above mentioned works are now cited in the revised version of the manuscript.
- Regarding efficiency of the method, the key message that we would like to convey with the paper is the difference in efficiency between filtering-based likelihood evaluation and the open loop approach and how the computational cost scales differently in the number of parallel inputs, measurement times, and number of cells. Our logic has thus been that all methods that are based on iterative likelihood evaluation fall into the same class and have similar efficiency. For instance, whether the LNA or a particular moment closure method is used to propagate moments between measurement times in Algorithm 1 is practically irrelevant for computation time. That said, the additional references that the reviewer suggested point to an aspect that we did not discuss in the original version of the manuscript: that computational efficiency depends on whether or not marginal likelihoods at measurement times can be calculated analytically. In response to the reviewer's comment, we have therefore added a discussion of this aspect to the novel paragraph in the Introduction.

I also want to mention that a variant of the Finite State Projection has also been used to compute the likelihood of single-cell trajectories in the following work:

• Andreychenko, L. Mikeev, D. Spieler, and V. Wolf, "Parameter Identification for Markov Models of Biochemical Reactions," in Computer Aided Verification, Berlin, Heidelberg, 2011, pp. 83–98. doi: 10.1007/978-3-642-22110-1_8.

An important similarity is that this work also restarted the CME approximation at each observation time and only used a small efficient FSP approximation that only needed to be valid during the short interval between two observation times. This work should be cited and compared with the new method advanced in the present manuscript.

- The work by Andreychenko et al. is now cited in the revised version of the manuscript.

Throughout section 3, the term "LNA" is used to refer to the author's new method which coupled moment closure, Gaussian approximation, and Kalman filtering as defined in

Algorithm 1. I find this confusing, and I suggest that the authors use a different name for their method.

- We have changed the terminology and now refer generally to iterative likelihood evaluation or Algorithm 1 to stress that the key ingredient for the accuracy of the results is the fact that likelihoods are evaluated by filtering rather than the particular method that is used to propagate moments between measurement time points.

It is not straightforward to me how The Finite State Projection is used in section 3.1.1. and Figure 2 to compute the single-cell likelihood. Did the authors compute the full dense matrix exponential of the FSP-truncated transition rate matrix? Or was it the much more efficient procedure described in Andreychenko et al. (comment 2 above)? In any case, I recommend a section is added to the supporting information to clearly explain the procedure used to obtain the FSP-based likelihood approximation in Figure 2.

- Yes, we have used just the normal matrix exponential without exploiting any particular structure for computational gains. The FSP approach was only used to generate a ground truth to compare to and there was no need to optimize its efficiency. We also point out that the case study is one-dimensional with fairly small molecule numbers. The generator matrix of the Markov chain could thus be truncated to comparably small size. If we understand the work by Andreychenko et al. correctly, the formulation of the problem that was considered in their paper was for a case where the complete state (i.e. all species) of the reaction network is observed. While this is also the case for our example in Figure 2 (since it is one-dimensional), the approach that we used to obtain the FSP-based likelihood is more general and works according to the same principles as Algorithm 1. To explain this, the Bayesian perspective of the filtering updates that need to be carried out at measurement times is most useful. What is used in Algorithm 1 (and in Kalman filtering) to ensure that state updates can be carried out analytically is the fact that a Gaussian prior is conjugate to a Gaussian likelihood and enables analytical calculation of the marginal likelihood.
  A second special case in which marginal likelihoods are tractable is when the prior lives on a finite state space, because in this case the marginal likelihood is a finite sum. FSP is an approach that yields a finite state space by construction. We have used this to generate the “ground truth” in Figure 2, basically by just plugging an FSP approximation instead of a moment-closure approximation or the LNA into Algorithm 1. Since this was not sufficiently explained in the original manuscript, we have added a section to the Supporting Information (Section B) where step 4 of Algorithm 1 is explained from a Bayesian perspective and the FSP version of the algorithm is discussed.
