# Supplementary material for: Parameter inference for stochastic biochemical models from perturbation experiments parallelised at the single cell level
Source: PLoS Comput Biol. 2022 Mar 18;18(3):e1009950. doi: 10.1371/journal.pcbi.1009950 (PMC8967023; doi:10.1371/journal.pcbi.1009950)
Supplement: Attachment — Submitted filename: Response.pdf [file pcbi.1009950.s004.pdf]

Point-by-point response to reviewers

Reviewer #3

Minor comments:

1. It appears to me the current title of the manuscript still seems to put the reader's focus more on the "parameter inference" part rather than the new experimental data type and empirical results that the authors aim to emphasize. For example, how about "New perturbation experiments parallelized at the single cell level enables efficient parameter inference for stochastic biochemical models"?

We have considered changing the title and thank the reviewer for coming up with a suggestion. All things considered, we have decided that it is best to keep the old title to avoid possible confusing of the published paper and the bioRxiv preprint.

2. On line 601, the authors claimed that "it has not been recognised that iterative likelihood evaluations may lead to vastly improved precision if the chemical master equation needs to be approximated". I have reservation about this claim, since it seems in much of literature on parameter inference of stochastic models that iterative approaches (such as particle filtering, particle marginal MCMC...) are the methods of choice when dealing with time-series data. The main question is what simulation/approximation of the CME (SSA, FSP, LNA...) is effective and efficient on the data at hand. Admittedly, when the data were sparse with long time intervals between measurements, many methods became either very slow or imprecise.

We have modified the corresponding sentence in the discussion section to avoid possibly debatable claims.
